# Juvenile vs. Adult-Onset Spondyloarthritis: Uncovering Similarities and Differences

**DOI:** 10.5152/ArchRheumatol.2026.25040

**Published:** 2026-04-03

**Authors:** Mehtap Kalçık Unan, Nebahat Zeynep Özaslan, Gamze Dilek, Nihal Şahin, Hafize Emine Sönmez, Kemal Nas

**Affiliations:** 1 Rheumatology Clinic, Diyarbakır Gazi Yasargil Training and Research Hospital, Diyarbakır, Türkiye; 2 Division of Pediatric Rheumatology, Department of Pediatrics, Kocaeli University, Kocaeli, Türkiye; 3 Bolu Abant İzzet Baysal University Faculty of Medicine, Bolu, Türkiye; 4 Division of Immunology, Rheumatology, and Algology, Department of Physical Medicine and Rehabilitation, Sakarya University, Sakarya, Türkiye

**Keywords:** Ankylosing spondylitis, enthesitis-related arthritis, peripheral arthritis, sacroiliitis

## Abstract

**Background/Aims:**

The primary objective of this investigation is to systematically assess the resemblances and distinctions between enthesitis-related arthritis (ERA), which serves as a pediatric analog to ankylosing spondylitis (AS), and AS.

**Materials and Methods:**

A cross-sectional analysis was conducted within a pediatric rheumatology facility and an adult rheumatology institution. Demographic and clinical attributes were juxtaposed between individuals diagnosed with AS and those with ERA.

**Results:**

A cumulative total of 200 subjects (101 minors, 99 adults) were scrutinized. The prevalence of peripheral arthritis was significantly elevated in ERA (54.5%) compared to AS (21.2%, *P* < .001), whereas sacroiliitis, axial involvement, and morning stiffness were more prevalent in AS. Non-steroidal anti-inflammatory drugs constituted the predominant initial therapeutic approach (99% vs. 91.9%, *P* = .01). Patients with ERA exhibited a higher utilization of corticosteroids (35.6% vs. 11.1%, *P* < .001) and disease-modifying antirheumatic drugs (DMARDs) (87.1% vs. 60.6%, *P* < .001), with no notable variance in the application of biological agents. At the final follow-up assessment, 24.8% of ERA patients were free from medication, whereas no AS patients achieved a medication-free status.

**Conclusion:**

An increased volume of scientific evidence is requisite concerning the attributes of ERA, which is presently regarded predominantly within the framework of juvenile spondyloarthritis, distinguishing it from its adult counterpart; this study serves as an observational cohort investigation that underscores this imperative. Despite the observable differences and similarities between ERA and AS, further extensive studies incorporating inflammatory biomarkers are essential to ascertain whether these conditions represent discrete diseases or exist within a broader disease continuum.

**Main Points**

- This study performed a comprehensive comparison of the clinical features, treatment approaches, and imaging findings of patients with enthesitis-related arthritis (ERA) and ankylosing spondylitis (AS) in the Turkish population.
- The analysis revealed that ERA is characterized by more peripheral joint involvement and a higher frequency of steroid and disease-modifying antirheumatic drugs use.
- The AS was associated with more prominent axial and neck involvement, prolonged morning stiffness, radiological progression, HLA-B27 positivity, and higher prevalence of uveitis.
- This study demonstrates that ERA and AS exhibit different clinical courses and treatment requirements.

## Introduction

The term spondyloarthritis (SpA) refers to a group of interrelated diseases characterized by various features, including arthritis affecting peripheral and axial joints, enthesitis, acute and painful anterior uveitis, and human leukocyte antigen (HLA) B27 positivity. This group includes ankylosing spondylitis (AS), the most typical form, along with reactive arthritis or Reiter’s syndrome, enteropathic arthritis, and psoriatic arthritis, with many overlapping clinical features among them.^1^ The onset of the disease occurs across a wide age range from childhood through adolescence to adulthood, with its incidence peaking in late adolescence and early adulthood.^2^ In adults, SpA diagnosis is established according to the Assessment of SpondyloArthritis International Society (ASAS) criteria. With these criteria, axial SpA (axSpA) can be diagnosed in patients who have chronic low back pain lasting more than 3 months and an onset before age 45 years, either by the presence of HLA-B27 and 2 clinical SpA features or by evidence of sacroiliitis on imaging (magnetic resonance imaging (MRI) or X-ray) along with 1 clinical SpA feature.^3^ Patients with sacroiliitis detected on X-ray are classified as radiographic axSpA, while those without definitive radiographic sacroiliitis are classified as non-radiographic axSpA. The most common form of axSpA includes AS.^4^ In the childhood classification criteria, enthesitis-related arthritis (ERA), a subgroup of juvenile idiopathic arthritis (JIA) based on the International League of Associations for Rheumatology (ILAR) criteria, is used as a substitute for AS, although it does not have an exact one-to-one correspondence.^5^ To diagnose ERA, there must be either the presence of both arthritis and enthesitis or 2 of the following criteria in the presence of arthritis or enthesitis: sacroiliitis, HLA-B27 positivity, family history, anterior uveitis, or being a male child over the age of 6. The classification criteria for ERA have been revised over time, with criticism directed at the current ILAR-based juvenile SpA (JSpA) criteria for not encompassing the full spectrum of SpA.^6^ In response, the Paediatric Rheumatology International Trials Organisation (PRINTO) proposed updated JIA criteria in 2019. Notable changes include raising the upper age limit for JIA from 16 to 18 years and renaming ERA to “enthesitis/spondylitis-related arthritis.”^7^ Furthermore, psoriatic arthritis is no longer classified separately, and sacroiliitis detected on imaging is now included as a diagnostic criterion.^7^ It has been suggested that early stages of spinal involvement, before radiographic findings develop, should be included to identify more homogeneous patient groups across age ranges, potentially improving treatment development.^7^ Additionally, there is no consensus on whether the disease represents age-related forms or a continuous spectrum with shared pathogenesis. The ERA has an incidence ranging from 13.4% to 3.4%, and its diagnosis is particularly challenging in younger paKtients, complicating classification in children and adolescents.^8^

Diseases that begin in childhood may differ in course, severity, and treatment response from those that manifest in adulthood, often requiring more specialized approaches. Few studies comprehensively compare clinical, laboratory, and treatment responses between childhood and adult-onset SpA, with ethnic variations influencing disease progression.^9^^-^^11^ Although ERA and AS are recognized as diseases with similar etiological and pathophysiological characteristics, the developmental disparities between pediatric and adult immune systems may facilitate the disease’s manifestation through the activation of distinct immunopathological pathways. This observation prompts significant inquiries regarding the interrelationship and pathogenesis of ERA and AS, which remain inadequately clarified in the existing literature. This prevailing knowledge deficiency constrains the ability to establish optimal diagnostic methodologies and to devise effective long-term management strategies that are appropriately tailored to both patient demographics. This study aims to summarize the clinical findings, treatments, and responses in patients with ERA and AS to highlight the similarities and differences between childhood versus adult-onset SpA. The objective is to provide insights into the inquiry regarding whether ERA and AS constitute components of a singular disease continuum or represent separate entities with notable distinctions, as well as to assist in the development of diagnostic and therapeutic strategies that are tailored to specific age groups.

## Material and Methods

In this cross-sectional study, data from patients who presented over a 6-month period were reviewed. To minimize data loss, patient information was recorded using standardized forms during follow-up visits.

The patients diagnosed with ERA according to ILAR criteria^5^ and AS according to ASAS criteria,^3^ who were referred to a tertiary pediatric and adult rheumatology center and followed, were included. Furthermore, the study encompassed participants who had a minimum of 12 months of follow-up evaluation. Criteria for exclusion comprised individuals with follow-up durations shorter than 12 months, those with incomplete data, and patients diagnosed with additional autoimmune or autoinflammatory disorders. A total of 200 patients were included in the study, comprising 101 diagnosed with ERA and 99 with AS. Demographic data, including age, sex, age at diagnosis, follow-up duration, comorbid conditions, and family history of rheumatic disease, were analyzed. Clinical features at diagnosis, such as peripheral arthritis, enthesitis, sacroiliitis, axial involvement, morning stiffness, and joint pain, were recorded. Enthesitis was defined as tenderness at the insertion point of a ligament or tendon on palpation. Morning stiffness duration was categorized as less than 15 minutes, 15-30 minutes, 30-45 minutes, 45-60 minutes, and over 60 minutes. Additionally, laboratory findings including HLA-B27 status, C-reactive protein (CRP) levels (with an upper limit defined as 5 mg/L), erythrocyte sedimentation rate (upper limit defined as 20 mm/hr), leukocyte count (×10^3^/µL), hemoglobin (g/dL), and platelets (×10^3^/L) along with treatment regimens and responses to treatment were assessed.

The Juvenile Spondyloarthritis Disease Activity Index (JSpADA) was used as the disease activity index for pediatric patients.^12^ For adult patients, the Bath Ankylosing Spondylitis Disease Activity Index (BASDAI) was used to assess disease activity.^13^ Based on the patients’ responses, each parameter was given a score between 0 and 10, with higher scores indicating more severe disease. Those with a BASDAI score of ≥ 4 were considered active patients. The sacroiliac joints on MRI were analyzed according to the recommendations of the Outcome Measures in Rheumatology.^14^ Information on Informed Consent: Before engaging in the study, every participant was enlightened about the process and granted both written and verbal approval.

The study was initiated after approval was obtained from the Ethics Committee of Kocaeli University (Approval number and date: KU GOKAEK-2024/10.24-274; June 11, 2024).

Radiographic sacroiliitis was defined based on 1984 modified New York criteria. Radiological progression was defined as erosion, syndesmophyte, and bridging syndesmophyte on cervical and lumbar spine radiographs.^15^

### Statistical Analysis

Statistical analyses were conducted using SPSS software, version 21 (IBM SPSS Corp.; Armonk, NY, USA). The variables were assessed using visual (histogram and probability plots) and analytical methods (Kolmogorov–Smirnov) to determine their distribution. Descriptive analysis data are presented as mean ± standard deviation or median (interquartile range) where appropriate. Categorical variables were compared using the chi-square test or Fisher’s exact test, as appropriate. The Student’s *t*-test or Mann–Whitney *U*-test was used to compare continuous data between the 2 groups. A multivariable logistic regression analysis was conducted to determine independent predictors of radiological progression. Age, sex, age at diagnosis, follow-up time, baseline disease activity, comorbidity, and HLA-B27 status were included as covariates. A sensitivity analysis was performed using a subcohort in which AS patients were matched to ERA patients with 18-24 months of follow-up, and the multivariable regression model was repeated to confirm that the findings were not influenced by differences in follow-up duration. Adjusted odds ratios (ORs) with 95% CIs were reported for each covariate. The goodness-of-fit and multicollinearity were evaluated before finalizing the model. A *P* value <.05 was considered statistically significant.

## Results

### Characteristics of Study Group

In this study, a total of 200 patients diagnosed with ERA and AS were evaluated, comprising 101 pediatric and 99 adult patients. For ERA patients, the median age at diagnosis was 13 years (8-16), and the median age at last follow-up was 16 years (9-21). For AS patients, the median age at diagnosis was 36 years (19-71), with a median age at last follow-up of 43 years (27-75). In terms of sex distribution, a male predominance was noted in both groups (62 in the ERA group and 70 in the AS group). Among ERA patients, the females were 39, males 62, whereas in AS patients, females were 29, males were 70. However, this difference did not reach statistical significance (*P *= .164). The median follow-up duration was 18 months (12-72) for ERA patients and 72 months (12-168) for AS patients. The prevalence of comorbid conditions was similar between the 2 groups, with 24.8% in ERA patients and 24.2% in AS patients (*P *= .933). A family history of rheumatic disease was reported in 35.6% of ERA patients and 23.2% of AS patients (*P* = .054). The mean body mass index was 25.87 ± 3.41 in AS patients and 20.75 ± 3.38 in ERA patients. The clinical findings and disease activity scores are shown in Table 1. In the multivariable logistic regression model, age emerged as a strong independent predictor of the outcome (OR = 1.22, 95% CI: 1.11-1.35, *P* < .001). Sex was not significantly associated with the dependent variable (*P* = .134). A higher age at diagnosis independently reduced the risk, with each 1-year increase corresponding to a 16% decrease in the odds of the outcome (OR = 0.84, 95% CI: 0.76-0.94, *P* = .003). The HLA-B27 status did not show significant associations in the adjusted model (overall *P* = .610). Among the activity scores, high disease activity demonstrated a borderline protective effect (OR = 0.27, 95% CI: 0.08-0.98, *P* = .046). The presence of an additional comorbidity was also not associated with the outcome (OR = 1.27, *P* = .573). A full summary of the regression coefficients is provided in Table 2.

The presence of peripheral arthritis, hip pain, and heel pain, as well as the number of active joints and enthesitis count, were more common in the ERA group, whereas sacroiliitis, axial involvement, neck pain, and uveitis were more common in the AS group. Although not reaching statistical significance, enthesitis was more common in ERA patients. Furthermore, the duration of morning stiffness was longer in AS patients. In the ERA group, the median disease activity score, measured using the JSpADA scale, was 3.6 (1.5-6) at the time of diagnosis. In comparison, the median BASDAI score for AS patients at diagnosis was 4 (1-5) (Table 1).

### Laboratory and Radiologic Findings

**Figure 1.**
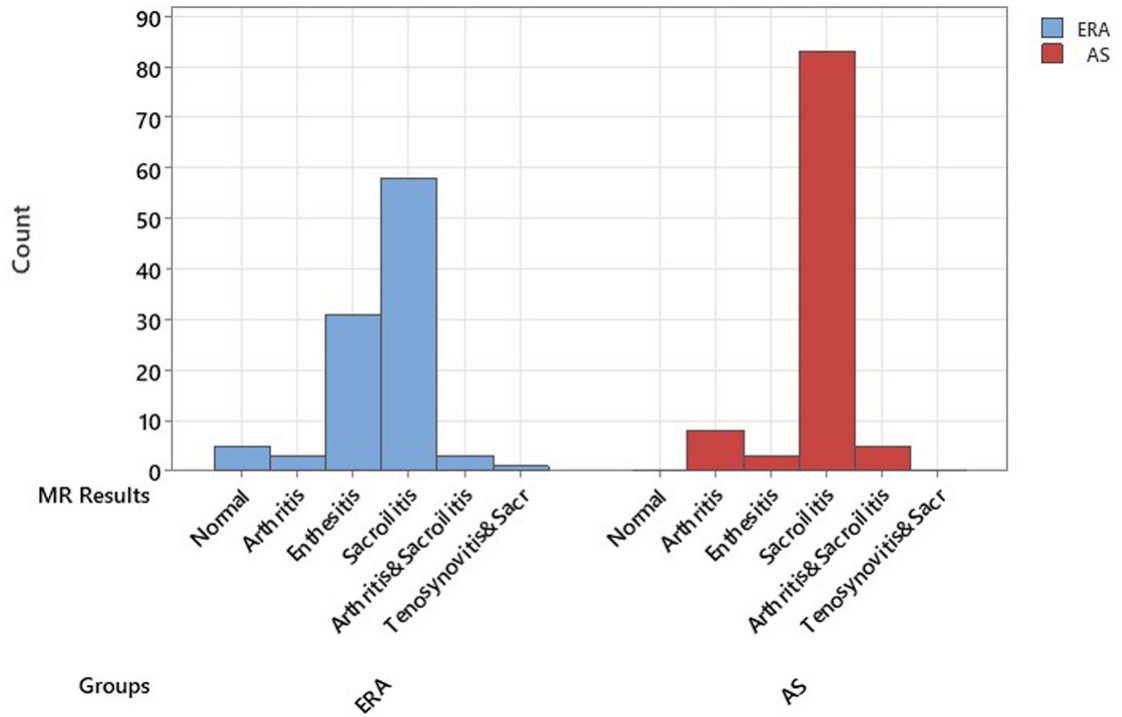
Comparison of MRI findings and radiological involvement in the ERA and AS group.

Laboratory values at diagnosis are given in Table 2. Leucocyte and platelet counts were higher in the ERA group, whereas CRP levels were higher in the AS group. The HLA-B27 positivity was higher in AS (69.8%) than in ERA (29.4%). No relationship was found between HLA-B27 positivity and clinical involvement such as peripheral arthritis, uveitis, axial involvement, enthesitis in the correlation analyses in the AS and ERA groups. When MRI findings at the time of diagnosis were analyzed, sacroiliitis was the most common finding, detected in 58 patients (57.4%) with ERA and 83 patients (83.8%) with AS (*P* < .001) (Table 3). The rate of non-radiological axial SpA (nr-axSpA) in adult SpA patients was found to be 21.7%. In the ERA group, 96.9% of patients with sacroiliitis detected by MRI (Figure 1a and b).

### Treatments

**Figure 2.**
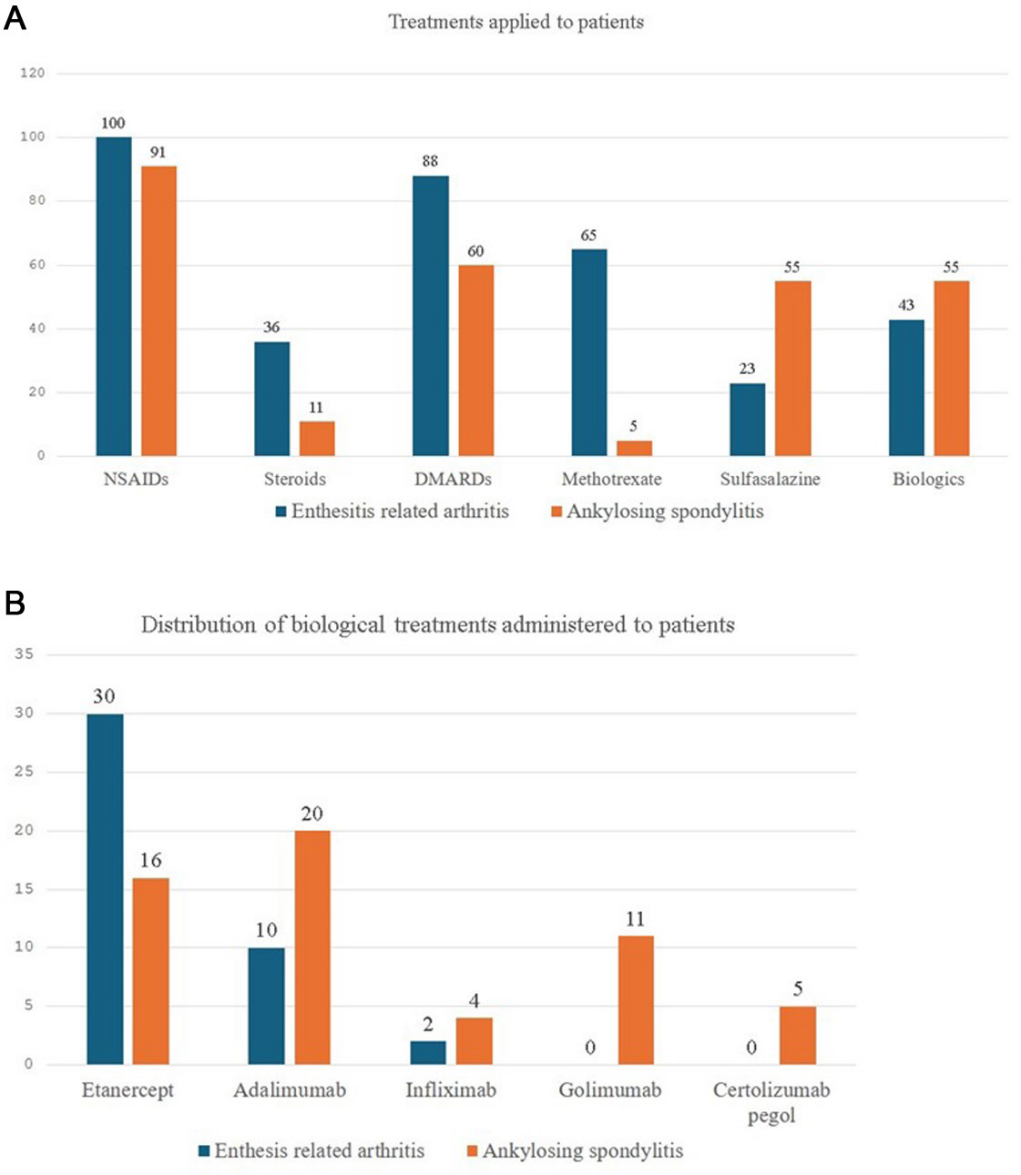
A) Treatments applied to patients diagnosed with enthesitis related arthritis and ankylosing spondylitis. B) Distribution of biological treatments administered to patients diagnosed with enthesitis-related arthritis and ankylosing spondylitis.

When examining treatment approaches, non-steroidal anti-inflammatory drugs (NSAIDs) were the most common initial treatment in both ERA and AS patient groups (99% vs. 91.9%, *P* = .01). The utilization of steroids (35.6% vs. 11.1%, *P* < .001) and disease-modifying antirheumatic drugs (DMARDs) (87.1% vs. 60.6%, *P =* .001) was significantly more frequent among ERA patients compared to AS patients. Meanwhile, no statistically significant difference was observed between the groups regarding the use of biologic agents (43.9% vs. 55.6%, *P *= .101) (Figure 2a).

In the ERA group, 65 patients (73.9%) were treated with methotrexate (MTX), while 23 patients (26.1%) received sulfasalazine. In contrast, in the AS group, only 5 patients (8.3%) used MTX, whereas 55 patients (91.7%) were treated with sulfasalazine. The median duration of DMARD use was 9 months (3-24) in the ERA group and 24 months (3-132) in the AS group. The DMARDs were switched to another agent in 14 patients (15.9%) in the ERA group compared to 2 patients (2.5%) in the AS group. Biological treatments were initiated in 43 ERA patients (42.5%) and 56 AS patients (56.5%). The median time to initiate biological therapy after the diagnosis was 6 months (3–24) in the ERA group and 24 months (3-36) in the AS group. In the ERA group, 15 patients (34.9%) used DMARD and biological therapy concomitantly, while in the AS group, 9 patients (16.1%) received both treatments. The DMARD therapy was discontinued within 3 months (1-12) of initiating biological treatment in 20 patients (11 from the ERA group and 9 from the AS group). When analyzing the distribution of biological agents used among patient groups, ERA patients demonstrated a significantly higher use of etanercept compared to AS patients (69.7% vs. 28.6%, *P* = .001). In contrast, AS patients showed greater usage of adalimumab (23.3% vs. 35.7%, *P* = .05), infliximab (4.7% vs. 7.1%, *P* = .124), golimumab (0% vs. 19.6%, *P* = .001), and certolizumab pegol (0% vs. 8.9%, *P *= .019) (Figure 2b). Biological drugs were switched to another agent in 9 ERA patients (20.9%) and 18 AS patients (32.1%). The first biological agent switch was made on average (10-24) months after the start of biological treatment in the AS group and (3.7-22.5) months in the ERA group.

### Outcomes

**Figure 3.**
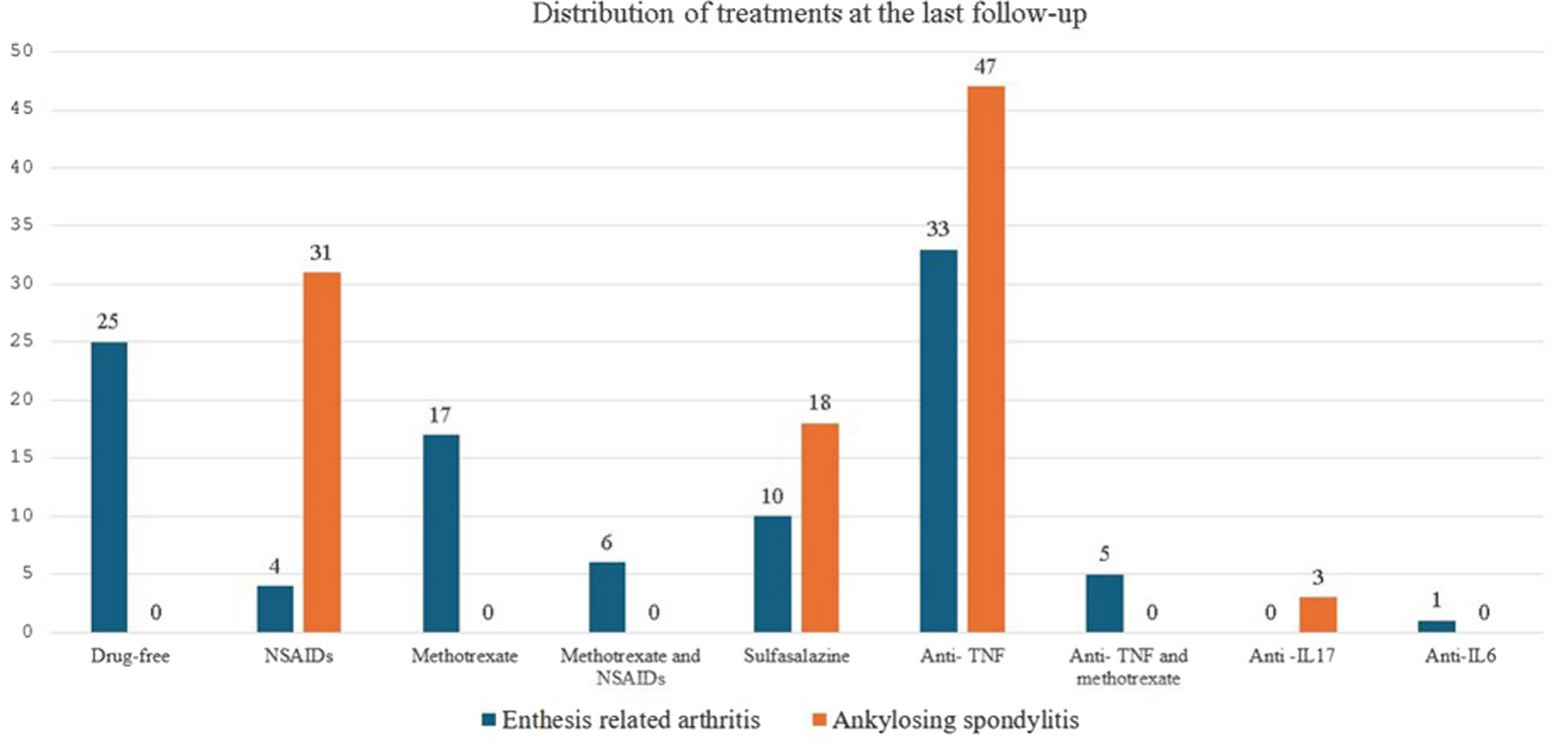
Distribution of treatments received by patients diagnosed with ERA and AS.

At the last follow-up, the median JSpADA score in the ERA group was 0.5 (0-4), while the median BASDAI score in the AS group was 2 (1-5). There was no significant difference in acute phase reactants at the last follow-up. In the ERA group, 24.8% of patients were followed without medication, whereas no patients in the AS group were managed without medication. In both groups, the most used treatment was anti-tumor necrosis factor (TNF) therapy (Figure 3). Radiological progression was observed in 11 (10.9%) ERA patients, while 54 (54.5%) AS patients showed radiological progression (*P* = .001). Disease activity scores, laboratory results, and treatments at the last follow-up are summarized in Table 4.

## Discussion

This study conducted a comprehensive comparison of the demographic characteristics, clinical features, laboratory parameters, treatment approaches, and imaging findings of patients with ERA and AS within the Turkish population. The analysis revealed that ERA was characterized by greater peripheral joint involvement and a higher frequency of steroid and DMARD use. In contrast, AS was associated with more pronounced axial and neck involvement, prolonged morning stiffness, radiological progression, HLA-B27 positivity, and a higher prevalence of uveitis. Despite these differences, both groups demonstrated similarities in the presence of comorbid conditions, male predominance, levels of acute phase reactants, and the use of biological agents in treatment. The age of diagnosis for ERA has been reported as 9.5-13 years in various studies.^2^ In the present study, the mean age at diagnosis for ERA was 13 years (8-16). Unlike JIA, which is more common in girls, ERA shows a male predominance, with reported male-to-female ratios ranging from 1.4:1 to 9:1.^8^ It is emphasized in the literature that gender is a demographically important risk factor in AS disease, as in ERA. Male superiority is observed in terms of incidence.^16^ In this study, there is a male dominance in ERA and AS patients, parallel to the literature. While the mean age at AS diagnosis was reported as 30.1 years in Stone et al’s study,^18^ it was 36 years (19-71) in the cohort. Male predominance was also more pronounced in adults, with a male-to-female ratio of 2.4:1. A Chinese study of 776 patients found a higher proportion of males in juvenile-onset SpA compared to adult-onset SpA (66.7% vs. 53.9%).^10^ However, no significant sex difference was observed between ERA and AS groups. In some publications, it has been reported that gender is genetically associated with HLA-B27 positivity, and it has been found to be higher in young men than in women.^17^ In this study, no effect of gender on disease onset age, HLA-B27 positivity, or family history was detected in the ERA and AS groups. The same Chinese study reported a higher family history of SpA in juvenile-onset cases compared to adults (23.7% vs. 15.4%).^10^ In the present study, family history rates were similar between juvenile and adult groups (35.6% vs. 23.2%), suggesting its importance across both groups.

Peripheral joint involvement and enthesitis are reported to occur more frequently in JSpA compared to adult-onset SpA.^9^^-^^11^ Huang et al^10^ observed higher rates of peripheral arthritis (83.3% vs. 73.6%) and enthesitis (35.1% vs. 23.3%) in JSpA. Similarly, Stone et al^18^ reported that JSpA patients were more likely to present with peripheral joint symptoms than other SpA forms (46.6% vs. 33.2%, *P* = .001). Another study found that 77.6% of JSpA patients presented with lower extremity arthritis, and 58.9% had hip arthritis at diagnosis.^19^ In the present study, consistent with the literature, peripheral joint involvement was significantly more common in pediatric patients (54.5%) than in adults (21.2%). Although the difference did not reach statistical significance, enthesitis was observed more frequently in patients with ERA (59.4% vs. 46.5%).

The frequency of axial skeleton involvement in children with JSpA has been reported to range from 28% to 78%^19^^-^^22^ with axial arthritis often developing in the later stages of the disease. Studies indicate that 30%-60% of children with ERA develop sacroiliac and/or spinal inflammation within 5-10 years after disease onset.^21^^-^^25^ In AS patients, high rates of sacroiliitis have also been reported.^18^^,^^26^ In this study, axial involvement was detected in 98% of AS patients and 64.4% of ERA patients, with sacroiliitis being more frequent in AS (89.9%) compared to ERA (62.4%). In the study, the rate of non-radiological axial SpA (nr-axSpA) in adult SpA patients was determined as 21.7%. In a cross-sectional study including 3984 SpA patients, the rate of nr-axSpA was reported as 14.4% in Asian countries and 24.3% in European, American, and African countries.^27^ In this study, inflammatory low back pain was similar in both groups, and hip pain was more common in the ERA group. However, 96.9% of ERA patients with sacroiliitis detected by MRI. While sacroiliitis is expected to be less common in ERA due to its later onset, some studies highlight the potential for earlier axial involvement. For instance, in an Italian cohort of 59 ERA patients, 35% reported inflammatory back pain within 15 months of disease onset, and 29% showed sacroiliitis on MRI. Similarly, in a multicenter cohort of 234 North American and European children with ERA, 25% presented with sacroiliac tenderness at diagnosis, and 55.6% of those undergoing MRI showed sacroiliitis.^25^ In this study, the lower rate of inflammatory back pain and the shorter follow-up duration for juvenile patients (18 months) may explain the reduced imaging frequency and detection of axial involvement. Children, being more active than adults, may experience less inflammatory back pain, which typically worsens with rest and improves with exercise. Given the importance of early detection of axial disease for optimal treatment, MRI is a valuable screening tool, particularly in asymptomatic children or adolescents with ERA. Notably, 20%-40% of cases with spinal involvement can progress asymptomatically and are only detectable via MRI.^28^^,29^ Furthermore, Demir et al^29^ demonstrated that 77% of asymptomatic ERA patients exhibited findings suggestive of axial involvement on spinal MRI. More frequent use of radiological methods is likely to influence and potentially alter diagnostic and therapeutic approaches.

The HLA-B27 is strongly associated with SpA and JSpA, with HLA-B27:05 being the most common allele across all populations.^30^ In a study of 135 ERA and 121 AS patients, HLA-B27 positivity was observed in 79% and 84%, respectively, with HLA-B27:05 more frequent in ERA (70% vs. 57%) and HLA-B27:04 less frequent (21% vs. 36).^31^ However, a Chinese study found no significant differences in HLA-B27 positivity between groups.^10^ In the present study, HLA-B27 positivity was higher in AS (69.8%) than in ERA (29.4%), aligning with the role of HLA-B27 in axial involvement, late disease onset, hip arthritis, and inflammatory markers.^24^^,^^32^^,^^33^ In the study, no relationship was found between HLA-B27 positivity and clinical involvement such as peripheral arthritis, uveitis, axial involvement, and enthesitis in the correlation analyses in the AS and ERA groups. Subtyping could not be performed due to incomplete testing.

Spondyloarthropathies can cause significant functional impairment and long-term consequences, making early inflammation suppression and prevention of adverse outcomes the primary treatment goals. The 2019 ACR JIA guidelines recommend NSAID monotherapy for enthesitis or sacroiliitis.^34^ Goirand et al^21^ reported inactive disease in 45.6% of cases treated with NSAIDs, which are effective for symptom relief but insufficient to prevent long-term damage. Consequently, NSAIDs are advised as bridging or adjunctive therapy rather than long-term monotherapy.^35^ In the present study, all ERA patients were started on NSAIDs at diagnosis, with 91.9% of adults also receiving NSAIDs. The DMARDs were used in 87.1% of pediatric and 60.6% of adult patients. Methotrexate was the most used first-line DMARD in ERA patients (73.9%), while sulfasalazine was preferred in 91.7% of AS cases due to its effectiveness in axial disease, where MTX has shown limited efficacy.^35^

For children with sacroiliitis unresponsive to NSAIDs, ACR guidelines recommend anti-TNF agents over MTX monotherapy.^34^ Anti-TNF agents, including etanercept, adalimumab, and infliximab, are effective in treating peripheral arthritis, enthesitis, and axial disease in ERA.^33^ These agents also reduce the development of anti-drug antibodies when combined with MTX. Although etanercept is the most used anti-TNF agent in practice, adalimumab is preferred for patients with uveitis due to its superior efficacy in uveitis and inflammatory bowel disease.^36^^-^^38^ In this study, etanercept was the most used biologic in ERA patients, while adalimumab was preferred in AS patients. Combined DMARD therapy was more frequent in ERA patients (34.9%) than in AS patients (16.1%). Ankylosing spondylitis patients had a higher rate of biologic switching (28.6%), likely due to greater availability of alternatives. Earlier studies, conducted before anti-TNF agents were introduced, reported longer diagnostic delays, less axial involvement, and more frequent uveitis and peripheral joint involvement in childhood-onset SpA.^9^ Outcomes differ significantly between patients with ERA and those with AS. Studies have shown that JSpA is associated with worse functional outcomes, often influenced by socioeconomic factors such as low income and younger age at onset.^18^ In the cohort, the rate of biologic agent switching tended to be higher in AS patients (32.1%) than in ERA patients (20.9%). However, because the statistical power of this subgroup analysis was limited, this finding needs to be confirmed in larger studies.

Additionally, patients with JSpA are more likely to require hip arthroplasty compared to adult patients.^39^^,^^40^ Beyond clinical assessments, evaluating radiological progression is crucial in understanding disease trajectory. Notably, baseline severity of MRI-evident sacroiliitis and HLA-B27 positivity in patients with early inflammatory back pain have been identified as predictors of radiographically evident AS after 8 years.^41^ Kim et al^42^ reported that radiographic progression over 5 years was significantly slower in juvenile-onset disease compared to the adult form. In the present study, radiological progression was observed in 54.5% of AS patients and 10.9% of ERA patients, which may be attributed to either the shorter follow-up duration in the ERA group or differences in the natural course of the diseases. The early clinical involvement of ERA patients, predominantly with peripheral involvement, and the longer it takes for the findings to become radiologically visible, even if symptomatic, may affect the results.

### Limitations

The primary limitation of our study was the lack of standardized outcome measurements for all age groups, combined with differences between adult and pediatric classification criteria, which makes comparisons quite challenging. Furthermore, the differences in long-term follow-up durations and the fact that treatment regimens were based on the clinicians’ experience are limitations of the study. Another constraint of our investigation is that MRI was not uniformly applied across all participants. In this cross-sectional analysis, which mirrors authentic clinical practice, the determination to conduct MRI was predominantly predicated on the clinician’s diagnostic suspicion (for instance, the manifestation of inflammatory low back pain). This could have engendered bias, particularly among patients exhibiting ERA who may lack axial symptoms yet may reveal radiological signs indicative of sacroiliitis. Also, a notable constraint of the investigation is the pronounced disparity in follow-up duration between the ERA cohort (median 18 months) and the AS cohort (median 72 months). This discrepancy primarily arises from the divergent typical ages of onset for these 2 conditions and the cross-sectional/retrospective design employed in the research; patients with AS were typically incorporated into the cohort characterized by a longer duration of the disease.

This study demonstrates that ERA and AS exhibit distinct clinical trajectories and therapeutic requirements. Juvenile patients are more frequently affected by peripheral involvement and enthesitis, whereas axial involvement and radiological progression are more predominant in AS patients. The potential value of MRI in detecting axial involvement in patients with ERA, particularly given the potential for asymptomatic progression, suggests that the role of this technique in monitoring radiological progression should be investigated in further prospective studies. These observed differences between ERA and AS highlight the need for age-specific management approaches and highlight the need for longitudinal studies to clarify the relationship between the 2 conditions and optimize long-term outcomes.

For better management of ERA disease progression, it is necessary to investigate the points that differentiate it from adult-onset SpA. The study will contribute to science in this context.

## Figures and Tables

**Table 1.** Demographic Data and Clinical Findings at the Time of Diagnosis in Patients Diagnosed with Enthesis-Related Arthritis and Ankylosing Spondylitis

	**Enthesis-Related Arthritis (n = 101)**	**Ankylosing Spondylitis (n = 99)**	*P*
Sex, Female/male	39/62	29/70	.164
Peripheric arthritis, n (%)	55 (54.5)	21 (21.2)	<.001
Enthesitis, n (%)	60 (59.4)	46 (46.5)	.089
Sacroiliitis, n (%)	63 (62.4)	89 (89.9)	<.001
Axial involvement, n (%)	65 (64.4)	97 (98)	<.001
Morning stiffness, n (%)	88 (87.1)	99 (100)	<.001
Duration of morning stiffness, n (%)			
<15 minutes	55 (62.5)	5 (5.1)	<.001
15-30 minutes	26 (29.5)	37 (37.4)	
30-45 minutes	3 (3.4)	36 (36.4)	
45-60 minutes	3 (3.4)	17 (17.2)	
>60 minutes	1 (0.9)	4 (4.04)	
Hip pain, n (%)	68 (67.3)	51 (51.3)	.03
Back pain, n (%)	38 (37.6)	37 (37.4)	.971
Neck pain, n (%)	4 (4)	18 (18.2)	.001
Heel pain, n (%)	45 (44.6)	26 (26.3)	.007
Uveitis, n (%)	0 (0)	7 (7.1)	.001
Active joint count, n (%)			
0	8 (7.9)	75 (75.8)	<.001
1-2	72 (71.3)	13 (13.1)	
>2	21 (20.8)	11 (11.8)	
Active enthesitis count, n (%)			
0	33 (32.7)	37 (37.4)	<.001
1-2	56 (55.4)	22 (22.2)	
>2	12 (11.9)	40 (40.4)	
BASDAI	NA	4 (1-5)	–
JSpADA	3.6 (1.5-6)	NA	–

*BASDAI, Bath Ankylosing Spondylitis Disease Activity Index; IQR, interquartile range; JSpADA, Juvenile Spondyloarthritis Disease Activity Index; SD, standard deviation.

**Table 2.** Variables Associated with the Radiologic Progression in Multivariable Logistic Regression

**Variable**	**Odds Ratio (OR)**	**95% CI for OR**	*P*
Age	1.222	1.105-1.352	**<.001**
Sex (Male)	0.533	0.234-1.215	.134
Age at diagnosis	0.844	0.755-0.944	**.003**
HLA-B27 (Positive)	1.417	0.591-3.398	.435
Comorbidity (Present)	1.270	0.553-2.918	.573
Disease activity (High)	0.273	0.076-0.977	**.046**

**Table 3.** Laboratory and Radiologic Findings at the Time of Diagnosis

		**Ankylosing Spondylitis**	*P*
Laboratory and radiologic findings			
Leucocyte, (×10^3^/ µL)	7.6 (4.3-26)	7 (3.8-15)	.05
Hemoglobin, (g/dL)	12.7 (6.24-16.1)	14 (8-16.4)	.002
Platelet, (×10^3^/L)	312 (171-745)	260 (138-573)	.001
Erythrocyte sedimentation rate, mm/hour	12 (2-81)	14 (1-66)	.123
C-reactive protein, mg/L	2.1 (0.16-64)	7 (3-65)	<.001
HLA-B27 positivity, n/N (%)	20/68 (29.4)	37/53 (69.8)	<.001
Magnetic resonance image findings			
Normal, n (%)	5 (5)	0 (0)	.025
Arthritis, n (%)	3 (3)	8 (8.1)	.113
Enthesitis, n (%)	31 (30.7)	3 (3)	<.001
Sacroiliitis n (%)	58 (57.4)	83 (83.8)	<.001
Arthritis and sacroiliitis, n (%)	3 (3)	5 (5.1)	.496
Tenosynovitis and sacroiliitis, n (%)	1 (1)	0	.321

HLA, human leukocyte antigen.

**Table 4.** Clinical Findings and Treatments Applied to Patients Diagnosed with Enthesis-relatedAarthritis and Ankylosing Spondylitis at the Last Follow-up

	**Enthesis-Related Arthritis**	**Ankylosing Spondylitis**	*P*
Uveitis, n (%)	0 (0)	8 (8.1)	.003
Active joint count, n (%)			
0	84 (83.2)	94 (94.6)	.02
1-2	16 (15.8)	4 (4)	
>2	1 (1)	1 (1)	
Active enthesitis count, n (%)			
0	80 (79.2)	66 (66.7)	<.001
1-2	21 (20.9)	13(13.1)	
>2	0 (0)	20 (20.2)	
BASDAI	NA	2 (1-5)	–
JSpADA	0.5 (0-4)	NA	–
Low disease activity, n (%)	100 (99.1)	35 (35.4)	<.001
High disease activity, n (%)	1 (0.9)	64 (64.6)	<.001
Erythrocyte sedimentation rate, mm/hour	8 (1-58)	8 (2-100)	.07
C-reactive protein, mg/L	1.05 (0.1-41)	4 (3-64)	<.001
Last visit treatment, n (%)			
Drug-free	25 (24.8)	0 (0)	<.001
NSAIDs	4 (3.9)	31(31.3)	<.001
Methotrexate	17 (16.8)	0 (0)	<.001
Methotrexate and NSAIDs	6 (5.9)	0 (0)	.040
Sulfasalazine	10 (9.9)	18 (18.2)	.090
Anti-TNF	33 (32.6)	47 (47.5)	.040
Anti-TNF and methotrexate	5 (4.9)	0	.020
Anti-IL17	0 (0)	3 (3)	.119
Anti-IL6	1	0 (0)	1
Radiological progression, n (%)	11 (10.9)	54 (54.5)	<.001

*BASDAI, Bath Ankylosing Spondylitis Disease Activity Index; JSpADA, Juvenile Spondyloarthritis Disease Activity Index; IL, interleukin; NSAIDs, non-steroid anti-inflammatory drugs; TNF, tumor necrosis factor.

## Data Availability

The data that support the findings of this study are available on request from the corresponding author.
